# Examining the Mediating Role of Motivation in the Relationship between Multidimensional Perfectionism and Well- and Ill-Being in Vocational Dancers

**DOI:** 10.3390/ijerph17144945

**Published:** 2020-07-09

**Authors:** Francisco L. Atienza, Isabel Castillo, Paul R. Appleton, Isabel Balaguer

**Affiliations:** 1 Department of Personality, Evaluation and Psychological Treatment, University of Valencia, 46010 Valencia, Spain; Francisco.L.Atienza@uv.es; 2 Department of Social Psychology, University of Valencia, 46010 Valencia, Spain; Isabel.Balaguer@uv.es; 3 School of Sport, Exercise and Rehabilitation Sciences, University of Birmingham, Birmingham B15 2TT, UK; p.appleton@bham.ac.uk

**Keywords:** perfectionism, motivation, well-being, ill-being, dancers

## Abstract

Perfectionism is considered to be an important personality factor within the dance context given the high number of dancers whose psychological health is influenced by its consequences. The relationship between perfectionism and dancers’ well- and ill-being can be mediated by a range of variables. The present study explores the role of forms of motivation (i.e., autonomous motivation, controlled motivation and amotivation) as mediators in the relationship between perfectionism (i.e., self-oriented and socially prescribed) and an indicator of well-being (i.e., subjective vitality) and ill-being (i.e., burnout). Participants of the study were 146 male and female Spanish vocational dancers aged between 12 and 26 years old (Mean age = 15.40 ± 2.96) who completed questionnaires measuring the variables of interest. Results of multiple mediator regression analyses showed that amotivation mediated the relationships between self-oriented and socially prescribed perfectionism with burnout and subjective vitality. Self-oriented perfectionism was negatively correlated, and socially prescribed perfectionism positively associated with amotivation. Amotivation of dancers was a positive predictor of burnout and a negative predictor of subjective vitality. Overall, the findings corroborate the importance of amotivation in the relationship between perfectionism dimensions and well-being and ill-being in dancers.

## 1. Introduction

Since some dancers are in constant pursuit of perfection, the dance domain is an ideal forum for the study of perfectionism [1]. Although there is a fairly general conception that perfectionistic dancers are vulnerable to maladaptive patterns of motivation, poor psychological health, and underperformance [2], it is also argued that dancers’ perfectionism can be positive when it drives them to achieve excellence [3]. Facing conflicting perspectives on whether dance perfectionism favors or hinders dancers’ optimal functioning, there is research interest whether this personality disposition in the discipline of dance may produce well- or ill-being, or both, in the most committed dancers [4,5].

It is widely accepted that perfectionism is a multidimensional personality characteristic. Multiple conceptual models have been developed to guide the research on perfectionism [6,7,8], with one of the most popular models originally proposed by Hewitt and Flett [7]. Hewitt and Flett [7] proposed that perfectionism encompasses intraindividual and interpersonal components of perfectionism and differentiate three major dimensions: (1) self-oriented perfectionism; (2) socially prescribed perfectionism; and (3) other-oriented perfectionism. Self-oriented perfectionism refers to the requirements for the self to be perfect and comprises beliefs that striving for perfection and being perfect is important. Socially prescribed perfectionism involves perceptions that significant others require the self to be perfect. This dimension comprises beliefs that others have excessively high standards for oneself and concerns, because of the belief that the acceptance by others is conditional on fulfilling these standards. Finally, other-oriented perfectionism refers to the belief that others should be perfect and is characterised by punitive criticism of others when they make mistakes.

In sport, previous research concerning well- and ill-being that has adopted Hewitt and Flett’s approach have generally focused on self-oriented perfectionism and socially prescribed perfectionism. The few studies in sport that have analyzed other-oriented perfectionism found that this dimension is principally associated with interpersonal difficulties, rather than personal maladjustment [9,10]. Socially prescribed perfectionism is considered a debilitating disposition, and in the sport context this dimension is consistently associated with lower levels of positive affect, subjective vitality, and life satisfaction [11], and higher levels of burnout [12,13].

Concerning self-oriented perfectionism, Hewitt and Flett [7] conceptualised this perfectionism dimension as a vulnerability factor that is associated with higher ill-being and lower well-being when the individual experiences stress, set-backs and failure. Some support for the maladaptive nature of self-oriented perfectionism is available in previous sports research, via a negative association with self-acceptance, and positive correlations with a fear of failure [12,14] and obsessive passion [15]. Conversely, it is possible for self-oriented perfectionism to be associated with more positive outcomes (and lower levels of maladaptive outcomes) when this perfectionism dimension is examined in isolation from stressful conditions. This has been the case in the majority of previous research in the sport setting where using a cross-sectional design, self-oriented perfectionism has been positively associated with positive affect, subjective vitality, life satisfaction [11] and harmonious passion [15], as well as lower levels of burnout [12,16,17].

Few studies have examined self-oriented and socially perfectionism in dancers. However, a study by Eusanio, Thomson and Jacque [4] studied the relationships between these perfectionism dimensions, shame and self-concept in a small sample (*n* = 24) of university students selected from dance classes. The findings reported by Eusanio et al. revealed that dancers’ socially prescribed perfectionism scores were positively associated with feelings of shame and negatively associated with self-concept. The findings concerning self-oriented perfectionism were non-significant.

Several studies have also confirmed the relationship between related dimensions of perfectionism and indices of psychological health and self-evaluations (see [18], for a recent review). The study by Cumming and Duda [19], for example, examined the relationships between profiles of multidimensional perfectionism and indices of body-related concerns and psychological health in vocational dancers. The results obtained showed that dancers with pure personal standards perfectionism (reflected by high scores in personal standards, moderate scores in concerns over mistakes and low scores in doubts about actions) reported the highest levels of psychological health. The vocational dancers with mixed perfectionism (reflected by high scores in personal standard, concerns over mistakes and doubts about actions) and pure evaluative concerns perfectionism (reflected by low scores in personal standards, moderate scores in concerns over mistakes and high scores in doubts about actions) were associated with the highest levels of psychological distress.

A more recent study by Quested, Cumming and Duda [20] also examined in vocational dancers the relationships between profiles of multidimensional perfectionism and indicators of self-evaluations (self-esteem and body dissatisfaction). The dancers in the pure personal standards perfectionism profile reported significantly lower body dissatisfaction than evaluative concerns perfectionism and mixed perfectionism dancers. The pure personal standards perfectionism profile was also associated with significantly higher self-esteem than evaluative concerns perfectionism and mixed perfectionism profiles.

The relationship between multidimensional perfectionism and burnout in vocational and professional dancers has been also analyzed in a study by Nordin-Bates, Raedeke, and Madigan [21]. The results obtained in this study show that dancers with pure evaluative concerns perfectionism and dancers with mixed perfectionism reported the most frequent burnout symptoms. The dancers with pure personal standards perfectionism, in contrast, reported the lowest levels of burnout. Overall, a growing body of research has been dedicated to examining the relationships between various facets of perfectionism and personal maladjustment in dancers. In contrast, the association of perfectionism, and specifically self-oriented and socially prescribed perfectionism, with experiences of well-being has been analyzed to a much lesser extent.

In addition to examining the associations between self-oriented and socially prescribed perfectionism with indicators of ill- (and to a lesser extent, well-) being, recent research in sport and dance has been interested in understanding the possible mediational mechanisms that help explain these relationships. One theory that has informed previous research examining the mediators of perfectionism dimensions is self-determination theory (SDT) [22,23]. SDT postulates that an individual’s level of self-determined motivation is reflected by the extent to which behaviour is regulated by processes that are congruent with the self. This theory forwards the existence of five motivational regulations and the lack of motivation: (1) intrinsic motivation, (2) integrated regulation, (3) identified regulation, (4) introjected regulation, (5) external regulation, and (6) amotivation. These motivational regulations are situated along a continuum of motivation ranging from high to low self-determination. The highest degree of self-determined motivation is represented by intrinsic motivation, which occurs when a person participates in an activity due to his or her interest or enjoyment is inherent to the activity. Integrated regulation refers to behaviours that are completely congruent with one’s values and goals. Identified regulation is evident when the activity is considered personally important, although not inherently enjoyable. Introjected regulation concerns behaviours that are executed to avoid feelings of guilt or shame or to fortify one’s self-worth. External regulation is the least self-determined type of motivation. An externally regulated behaviour is engaged in for extrinsic, instrumental reasons (e.g., to receive external rewards, or to avoid fear of punishment). Finally, amotivation reflects an absence of motivation and is observed when individuals are engaged in activities without any intentionality to conduct those behaviours [24].

Ryan and Deci [24] suggest that the continuum of behavioural regulations represents two forms of motivation: autonomous and controlled motivation. Autonomous motivation comprises intrinsic motivation, integrated regulation, and identified regulation, whereas controlled motivation comprises introjected regulation and external regulation. Amotivation is not captured by autonomous nor controlled motivation, as it is to be without intention or motivation for a particular behaviour. This theory also considers that the forms of motivation may have diverse consequences on individuals’ well- or ill-being. Specifically, SDT proposes that autonomous motivation will lead to better psychological adjustment and well-being, while controlled motivation will lead to psychological imbalance and ill-being [25].

There is a broad field of research that has consistently shown the implications of the types of motivation for well- and ill-being in sport. High-quality motivation (intrinsic or autonomous motivation) has been repeatedly associated with indicators of well-being, whereas low quality (controlled motivation) and the absence of motivation (i.e., (amotivation) has been related with different indicators of ill-being [26]. Likewise, previous studies with vocational dancers revealed that autonomous motivation was positively related to indicators of well-being such as self-esteem, while controlled motivation and amotivation were linked positively with indicators of ill-being such as burnout or social physique anxiety [27,28].

The relationship between motivation and perfectionism has also been proposed [7,29] and supported in sport and dance research (e.g., [17,30]). Hewitt and Flett [7] originally argued that self-oriented perfectionism and socially prescribed perfectionism were associated with different types of motivation. Regarding the former, Hewitt and Flett [7] proposed that self-oriented perfectionism would be associated with autonomous and controlled motivation. The pursuit of internally-set achievement-related goals and the pursuit of self-development that characterise self-oriented perfectionism make an association with autonomous motivation likely. Equally, though, self-oriented perfectionism is also expected to be associated with controlled motivation as a result of the contingent self-worth and fragile self-esteem that characterises this perfectionism dimension [17].

Regarding socially prescribed perfectionism, Hewitt and Flett [7] indicated this perfectionism dimension will be related to non-self-determined extrinsic motivation and amotivation. They considered that socially prescribed perfectionism will be characterised by controlled motivation due in part, to the individual’s huge desire to gain others’ approval and avoid punishments. Socially prescribed perfectionism is also expected to be associated with amotivation because this perfectionism dimension encapsulates feelings of hopelessness when striving for externally-imposed standards that are perceived to be unattainable [31].

Research studying the association between Hewitt and Flett’s [7] perfectionism dimensions and motivation in sport have provided some support the hypothesised associations. Self-oriented perfectionism has emerged as a positive predictor of components of autonomous and controlled motivation [32,33,34], whereas socially prescribed perfectionism has been positively related to maladaptive patterns of motivation [32,33,35]. A more recent study with elite junior athletes [17] also reported a positive relationship between socially prescribed perfectionism and amotivation, external regulation and introjected regulation, but surprisingly positive associations with one component of autonomous motivation (identify regulation) was also found. Conversely, self-oriented perfectionism was positively related to indicators of autonomous motivation (intrinsic motivation) and controlled motivation (external regulations and introjected regulation) and negatively with amotivation [17].

In dance, the relationships between profiles of multidimensional perfectionism and indicators of motivation-related constructs (intrinsic motivation and fear of failure) were reported by Quested et al. [20]. The results showed that dancers with pure personal standards perfectionism reported higher intrinsic motivation than dancers with pure evaluative concerns perfectionism, but the dancers with mixed perfectionism did not exhibit higher levels of intrinsic motivation than either pure evaluative concerns perfectionism or pure personal standards perfectionism dancers. The pure personal standards perfectionism profile dancers had significantly lower fear of failure than evaluative concerns perfectionism and mixed perfectionism dancers.

Previous research in sport also provides insight into the mediating role of motivation regulations in the relationships between perfectionism and athlete burnout. Appleton and Hill’s study [17], for example, showed that amotivation mediated the positive relationship between socially prescribed perfectionism and some burnout symptoms, whereas amotivation and intrinsic motivation mediated the negative relationship between self-oriented perfectionism and burnout symptoms. The study of Jowett et al. [36] showed that autonomous and controlled motivation partially mediated the relationship between perfectionism and athletes’ burnout. Specifically, perfectionistic concerns showed a positive direct and indirect relationship with burnout, via controlled motivation. Perfectionistic strivings, in contrast, showed a negative direct and indirect relationship with burnout via autonomous motivation. Finally, a more recent study by Madigan, Stoeber, and Passfield [37] using a longitudinal design showed that autonomous motivation mediated the negative relationship between perfectionistic strivings and burnout, whereas controlled motivation mediated the positive relationship between perfectionistic concerns with burnout.

To date, as far as we know, no research has examined whether motivation regulations mediate the effects of perfectionism in dancers. However, previous authors [20] have proposed that dancers’ striving for perfectionism can be regulated by different motives, and according to these different motives, perfectionism has been associated with a range of adaptive and maladaptive outcomes. For some dancers, striving for a perfect performance may be challenging and moved by intrinsic motivation which is normally linked to positive psychological consequences, whereas other dancers need to perform perfectly is underpinned by feelings of fear, anguish, low-self-evaluation which subsequently contribute to negative psychological consequences [38,39].

Based on the postulates of SDT and the aforementioned research evidence outlined above, the main objective of the present study was to explore the role of forms of motivation (i.e., autonomous, controlled and amotivation) in the relationships between self-oriented and socially prescribed perfectionism and indicators respectively of well- (i.e., vitality) and ill- (i.e., burnout) being in a sample of young vocational dancers. Following previous literature evidence [11,13,36], we hypothesised that forms of motivation will act as mediators of the relationships between perfectionism and well- and ill-being. Specifically, we proposed the following hypotheses:

**Hypothesis** **1.** A positive association between socially prescribed perfectionism and burnout will be indirect via its (socially prescribed perfectionism) positive relationship with motivation variables (controlled motivation and amotivation).

**Hypothesis** **2.** The negative relationship between self-oriented perfectionism and burnout will be indirect through its (self-oriented perfectionism) positive relationship with autonomous motivation and negative relationship with amotivation.

**Hypothesis** **3.** The negative relationship between socially prescribed perfectionism and vitality will be indirect via positive relationships between this perfectionism dimension and the motivation variables (controlled motivation and amotivation).

**Hypothesis** **4.** The positive relationship between self-oriented perfectionism and vitality will be indirect via its (self-oriented perfectionism) positive relationship with autonomous motivation and negative relationship with amotivation.

## 2. Materials and Methods

### 2.1. Participants

The participants of our study were 146 male (*n* = 22) and female (*n* = 123) Spanish vocational dancers aged between 12 and 26 years old (M = 15.40, SD = 2.96) from two cities in Spain. Regarding their dance practice, 42.5% of the participants were involved in classical dance, 29.5% were in contemporary dance, 33.6% of the participants were involved in Spanish dance, and 6.2% of the participants were involved in different types of dance. They had been dancing for an average of 6.71 years (SD = 3.51) and spent 14.61 (SD = 5.26) hours dancing per week.

### 2.2. Instruments

The Spanish version adapted to dancers [40] of the Multidimensional Perfectionism Scale [7] was used to assess dancers’ perfectionism. The scale includes 10 items encompassing two subscales: (1) self-oriented perfectionism (e.g., “One of my goals is to be perfect in everything I do”), and (2) socially-prescribed perfectionism (e.g., “I find it difficult to meet others’ expectations of me”). Dancers respond to the scale on a 7-point Likert scale, ranging from 1 strongly disagree to 7 strongly agree. Evidence of the validity and reliability of the respondents’ scores in different contexts has been provided by previous studies [40,41].

Dancers’ motivation was measured using the Spanish version [42] of the Sport Motivation Scale [43], adapted for dancers. This instrument includes 28 items measuring five sub-dimensions: intrinsic motivation (twelve items; “For the pleasure it gives me to know more about the activity that I practice”); identified motivation (four items; “Because, in my opinion, it is one of the best ways to meet people”); introjected regulation (four items; “Because I must participate in dance to feel good about myself”); external regulation (four items; “Because it allows me to be well regarded by people that I know”); and amotivation (four items; “It is not clear to me anymore why I participate in dance”). The questionnaire begins with the stem “Why do you participate in dance?” and the items are answered on a 7-point Likert scale ranging from 1 (corresponds not at all with me) to 7 (corresponds exactly with me). The validity and reliability of respondents’ scores have been supported in previous studies focused on the sport context [17,42]. Intrinsic and identified regulations were used as indicators of autonomous motivation and introjected regulation and external regulation were used as indicators of controlled motivation.

Dancers’ psychological well-being was assessed with the Spanish version [44] of the Subjective Vitality Scale [45]. It is a six-item scale answered on a 7-point Likert scale ranging from 1 (not true) to 7 (true). Dancers were asked to consider the items in terms of whether they “generally” are true for them. An example item is ‘‘I feel alive and full of vitality’’. Previous studies have supported the reliability and validity of dancers’ scores on this scale [46].

To assess dancers’ ill-being, we used the Spanish version [47] of the 15-item Athlete Burnout Questionnaire [48] modified for the dance population. This scale contains five items sub-dimensions: emotional and physical exhaustion (i.e., “I feel overly tired from my dance participation”), degree of dance devaluation (i.e., I am not into dance like I used to be”), and reduced sense of dance accomplishment (i.e., “I am not achieving much in dance”). Responses are provided on a 5-point scale ranging from 1 (almost never) to 5 (almost always). For this study, we used a composite scale score reflecting global burnout. Evidence for the reliability and validity of dancers’ scores on this questionnaire has been reported [27,49].

The Cronbach alpha coefficients of the instruments are shown in Table 1.

### 2.3. Procedure

This research was conducted in accordance with international ethical guidelines that are consistent with the American Psychological Association guidelines, and with the Declaration of Helsinki. No additional approval was required from the Ethics Committee of the University of Valencia (Spain) because the data obtained do not involve health information. Since the study involved no invasive or potentially harmful elements, it was declared exempt of parent’s consent from review by the ethics committee. Participants were recruited from different professional dance schools from two Spanish cities. In all the schools, the researcher in charge of the study talked to the directors of the schools to explain the aim of the study and ask for permission for the administration of the questionnaire and to organise the data collection. Before the data collection, the researchers explained to the participants the objective of the study and indicated that their participation was anonymous and voluntary. They were asked to complete and sign an informed consent before completing the questionnaire.

### 2.4. Data Analysis

The IBM SPSS statistics 24 (IBM Corp., Armonk, NY, USA) was used to preliminary analysis, descriptive statistics, multivariate analysis of variance (MANOVA) analysis to examine types of dance differences (i.e., classical, contemporary and Spanish dance), gender and age group differences, internal consistency (Cronbach’s alpha), as well as the Pearson correlations between the study variables. We used Hayes [50] PROCESS macro for SPSS to determine whether the associations between dimensions of perfectionism and well- and ill-being variables were mediated by the types of motivation, and the bootstrapping method based on 1000 samples was applied to estimate standard errors of indirect effects. The indirect effects (ab) were calculated, where a1, a2 and a3 are the coefficients estimating the relationship between dimensions of perfectionism and types of motivation, and b1, b2 and b3 are the coefficients estimating the relationship between types of motivation and subjective vitality and burnout. If the BC bootstrap CI for the indirect effects (ab) does not include zero, mediation is supported.

## 3. Results

### 3.1. Preliminary Analysis

Prior to the main analyses, a missing value analysis was conducted on the data. Due to large amounts of missing data (>5%), 16 participants were removed from the sample. An inspection of the pattern of missing data suggested a non-systematic mechanism for the missing data. Consequently, the missing data were considered as missing at random and estimated using the Expectation Maximization Algorithm for analysis purposes. Following this procedure, the data was then screened for univariate outliers (standardised z-scores larger than 3.29, *p* < 0.001, two-tailed). Two participants showed outliers for amotivation items and were removed from the sample.

Analyses were based on 128 respondents who had reasonably complete data for the multidimensional perfectionism, motivational regulations, subjective vitality and burnout scales (i.e., they had missing values for no more than one item of the scales analyzed).

Results of MANOVA examining whether any differences existed between the three dance forms found no significant differences between them (Wilks’ lambda = 0.87, F (14,240) = 1.19, *p* = 0.29). Results also revealed that there were no significant gender and age differences (gender Wilks’ lambda = 0.93, F (14,270) = 0.66, *p* = 0.81; age Wilks’ lambda = 0.94, F (7,135) = 1.30, *p* = 0.25; age x gender Wilks’ lambda = 0.96, F (7,135) = 0.75, *p* = 0.63), and therefore subsequent analyses were conducted considering the whole sample.

### 3.2. Descriptive Statistics and Reliabilities

The dancers reported moderately high levels of self-oriented perfectionism and moderate levels of socially prescribed perfectionism. The dancers reported high levels of autonomous motivation, moderate levels of controlled motivation, and low levels of amotivation. Overall, dancers reported high levels of subjective vitality and low levels of burnout. Cronbach’s alpha coefficients were above 0.70 suggesting acceptable levels of internal reliability for each scale (see Table 1).

### 3.3. Relationships among the Study Variables

Self-oriented perfectionism was positively correlated with autonomous motivation and with subjective vitality, and negatively correlated with amotivation and with burnout. Socially prescribed perfectionism was positively correlated with controlled motivation, with amotivation and with burnout. Autonomous motivation was positively correlated with subjective vitality and negatively correlated with burnout. Controlled motivation was positively correlated with subjective vitality. Finally, amotivation was negatively correlated with subjective vitality and positively correlated with burnout (see Table 1).

### 3.4. Mediation Effect

To test whether motivation would mediate the relationships between the dimensions of perfectionism and well- and ill-being indicators, parameters for regression equations were estimated. Specifically, we examined perceptions of autonomous motivation, controlled motivation and perceptions of amotivation as mediators in the associations between a dimension of perfectionism (self-oriented or socially prescribed) and well- or ill-being (subjective vitality or burnout), using the other dimension of perfectionism as a statistical control variable. The corresponding perfectionism dimension that was used as control was introduced as a covariate in the model, hence estimating its effect on both the mediator and outcome variables.

**Figure 1:**
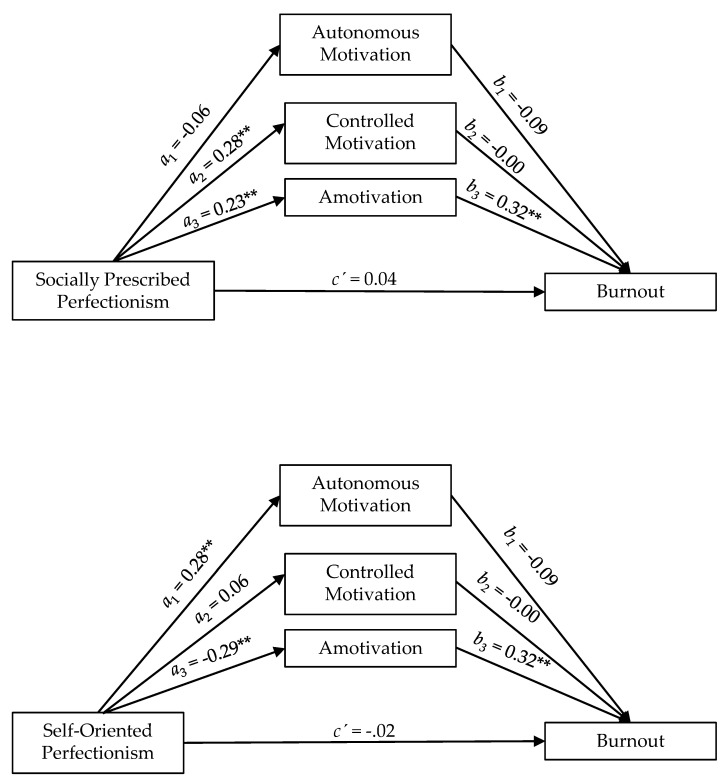
Graphic representation of forms of motivation as mediators between socially prescribed perfectionism and self-oriented perfectionism with burnout. ** *p* < 0.01; † *p* < 0.10.

**Figure 2:**
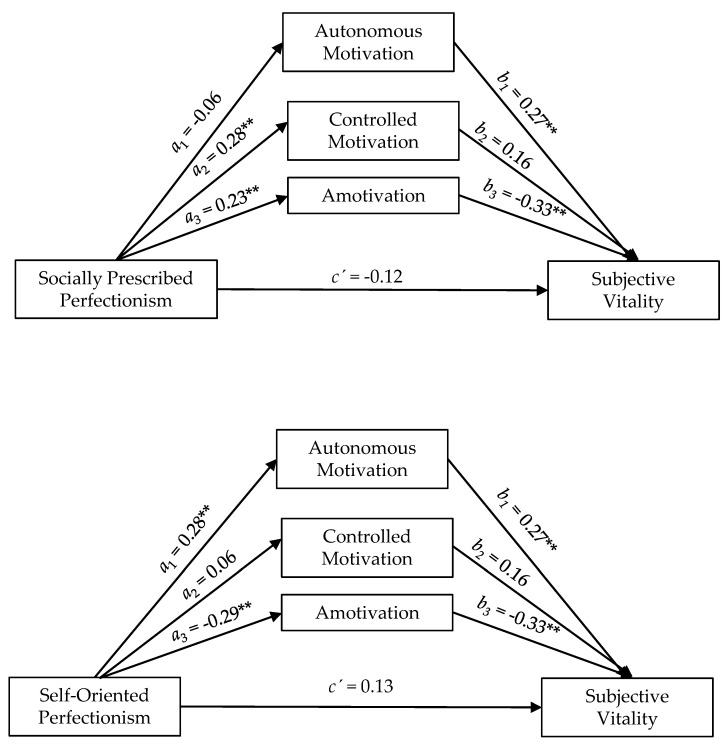
Graphic representation of forms of motivation as mediators between socially prescribed perfectionism and self-oriented perfectionism with subjective vitality. ** *p* < 0.01; * *p* < 0.05.

The results showed that socially prescribed perfectionism positively predicted controlled motivation (a_2_ = 0.28, *p* < 0.01), and amotivation (a_3_ = 0.23, *p* < 0.01), whereas self-oriented perfectionism positively predicted autonomous motivation (a_1_ = 0.28, *p* < 0.01) and negatively predicted amotivation (a_3_ = −0.29, *p* < 0.01). Autonomous motivation positively predicted subjective vitality (b_1_ = 0.27, *p* < 0.01) and negatively predicted burnout (b_1_ = −0.09, *p* < 0.10), whereas amotivation positively predicted burnout (b_3_ = 0.32, *p* < 0.01) and negatively predicted subjective vitality (b_3_ = −0.33, *p* < 0.01) (see Table 2 and Figure 1 and Figure 2).

As shown in Table 2, autonomous motivation and controlled motivation did not mediate the relationships between perfectionism dimensions and indicators of well and ill-being (confidence intervals included zero); however, the relationships between self-oriented perfectionism with subjective vitality and burnout were indirect via amotivation. Self-oriented perfectionism indirectly and positively predicted subjective vitality (IE = 0.10) and negatively predicted burnout (IE = −0.09) through its negative relationship with amotivation. Likewise, the relationships between socially prescribed perfectionism with subjective vitality and burnout were also indirect via amotivation. Socially prescribed perfectionism indirectly and negatively predicted subjective vitality (IE = −0.08) and positively predicted burnout (IE = 0.07) through its positive relationship with amotivation. The values of the relationships varied from medium and large sized effects [51] (see Table 2 and Figure 1 and Figure 2).

## 4. Discussion

Building upon previous research that has focused predominantly on the relationships between self-oriented and socially prescribed perfectionism with indicators of ill-being in sport, the purpose of this study was to explore the relationships between these perfectionism dimensions with burnout, as an indicator of ill-being, and with subjective vitality as an indicator of well-being in vocational dancers. We also analyzed the mediating role of motivation quality (autonomous motivation, controlled motivation) and amotivation, between each dimension of perfectionism (self-oriented and socially prescribed) and burnout and subjective vitality.

As expected, and consistent with previous cross-sectional research in sport [11,12,14,16,17], preliminary bivariate correlations revealed that socially-prescribed perfectionism was related to higher levels of burnout. These findings provide initial evidence regarding the potentially debilitating effects of socially prescribed perfectionism for dancers’ psychological health. However, given the lack of significant correlations between socially prescribed perfectionism and subjective vitality, future research may wish to measure additional indicators of well-being to provide further insight into the implications of this perfectionism dimension for dancers’ well-being.

Preliminary bivariate correlations also revealed that self-oriented perfectionism was related to lower levels of burnout and higher levels of vitality, and these findings are also consistent with previous cross-sectional research in sport. Specifically, the findings suggest that this perfectionism dimension appears somewhat beneficial for dancers’ psychological health when examined in the absence of stress-related factors. Further research is thus now required to extend our study, that examines whether the vulnerable nature of self-oriented perfectionism as originally proposed by Hewitt and Flett [7] emerges in dancers when they experience set-backs and failures as well as when exposed to stressful situations (e.g., dance exams; auditions). Such research should offer us more precise information regarding the conditions that render dancers vulnerable to the hypothesized negative implications of self-oriented perfectionism for their psychological health.

Our bivariate correlations also showed that self-oriented perfectionism was related to higher levels of autonomous motivation and with lower levels of amotivation, whereas socially-prescribed perfectionism was associated with higher levels of controlled motivation and amotivation. Hewitt and Flett [7] proposed that self-oriented perfectionism should be characterised by autonomous and controlled motivation, whereas socially prescribed perfectionism was expected to be associated with controlled motivation and amotivation. Our findings, are therefore, in partial agreement with Hewitt and Flett’s [7] conceptualization and suggest autonomous motivation may be one psychological mechanism that explains why self-oriented perfectionism has been associated positively with indicators of well-being in previous research, especially under certain non-stressful conditions. Conversely, the motivational profile associated with socially prescribed perfectionism explains why this perfectionism dimension holds debilitating implications for dancers’ psychological health.

The principal interest of the current study was to explore the mediating role of motivation in the relationship between self-oriented and socially prescribed perfectionism and indicators of well- and ill-being (i.e., subjective vitality and burnout) in a sample of young vocational dancers. Regarding the mediational effects concerning autonomous motivation and controlled motivation, results were contrary to our hypotheses. That is, the relationships between self-oriented perfectionism and socially-prescribed perfectionism with subjective vitality and burnout were not mediated either by the autonomous or controlled motivation of dancers. These results are partially consistent, however, with previous research involving athletes [17] in which it was found that the relationships between self-oriented perfectionism and socially prescribed perfectionism and burnout were not mediated by extrinsic motivation regulations. Appleton and Hill [17] proposed a possible explanation for their results, suggesting that introjected and external regulations (i.e., controlled motivation) may fail to capture the motivational signature of athlete burnout. In support of Appleton and Hill’s explanation, controlled motivation was not significantly correlated with burnout at the bivariate level in our study (although it did have a positive correlation with subjective vitality). Another possible explanation for the non-mediating role of autonomous motivation and controlled motivations may be related to the limitations associated to the measurement of motivation through the adapted Sport Motivation Scale for dance due to the ambiguity in some of the items and the difficulties in capturing the central hallmarks of non-self-determined extrinsic regulations [17].

Although autonomous and controlled motivation failed to mediate the hypothesised effects of self-oriented and socially prescribed perfectionism, it is worth reinforcing that self-oriented perfectionism was significantly and positively correlated with autonomous motivation and socially prescribed perfectionism was also significantly and positively correlated with controlled motivation at the bivariate level, providing some evidence of the motivational nature of self-oriented perfectionism and socially prescribed perfectionism beyond just amotivation. Thus, future research may wish to adopt alternative measures of dancers’ autonomous and controlled motivation, as well as additional indicators of well- and ill-being when re-examining the mediating role of motivation on the effects of self-oriented and socially prescribed perfectionism for dancer’s psychological health.

The results of the current study provided partial support for the hypothesised role of amotivation as a mediator of the relationships between self-oriented perfectionism and socially-prescribed perfectionism with burnout and subjective vitality of dancers. Specifically, the results confirmed the total mediation of amotivation suggesting that the negative relationship of socially prescribed perfectionism with subjective vitality and the positive relationship with burnout of vocational dancers were indirect through the positive relationship between socially prescribed perfectionism and amotivation. Likewise, the positive relationship between self-oriented perfectionism with subjective vitality and the negative relationship with vocational dancers’ burnout scores were also indirect through the negative association between self-oriented perfectionism on amotivation.

Concerning burnout, the present findings are consistent with previous research and confirmed the results of Appleton and Hill [17] indicating that this form of (lack of) motivation (i.e., amotivation) is also central to understanding the positive relationship between socially prescribed perfectionism and burnout in dancers. This dimension of perfectionism, where dancers try to attain standards and expectations prescribed by significant others, involves an external locus of control reflected by feeling that one’s achievement of desired standards are out of their control and thus never possible. Such feelings likely culminate in a sense of hopelessness that is captured by amotivation [31], which are subsequently reflected in emotional and physical states such as burnout.

Our results also confirmed the importance of this type of (lack of) motivation to explain the inverse relationship between self-oriented perfectionism and dancers’ burnout, as well as the positive relationship between self-oriented perfectionism and vitality, and replicate the findings reported by Appleton and Hill [17] in sport. Unlike socially prescribed perfectionism, it seems that internally set performance goals and desired standards that characterise self-oriented perfectionism may be sufficient to allow dancers to retain some ownership over achievement striving as well as, on occasions, to experience feelings of competence. As a result, it is less likely dancers high in self-oriented perfectionism will report amotivation (at least in the short-term) and it is well established that low levels of amotivation reduce the occurrence of burnout whilst simultaneously enhancing opportunities for feelings of vitality. Our findings provide initial support for this explanation in dancers.

## 5. Limitations

We consider that a strength of the present study is the fact that we were able to partially replicate the findings reported by Appleton and Hill [17], as well as extending their findings by including an indicator of well-being (i.e., subjective vitality). Despite this strength, several limitations existed in the current study. The study utilised a cross-sectional design, which does not capture the developmental aspects of the modeled relationships. Future research should reinvestigate these relationships longitudinally, which is especially important given the vulnerable nature of self-oriented perfectionism may emerge over time given dancers are likely to suffer setbacks, failures, and stresses. Another limitation is related to our sample of dancers. It may be considered relatively small and focused on vocational dancers with a greater proportion of female dancers. Future studies should recruit larger samples, employing a sample with a greater proportion of male dancer and reinvestigate the relationships analyzed examining also whether the findings generalise to professional dancers.

## 6. Practical Implications

The findings of the current study have a number of practical implications. In particular, the findings suggest that interventions are needed to prevent the occurrence of (or reduce already established) socially prescribed perfectionism and the associated lack of motivation for dancers. Whilst research in other domains has pointed towards the benefits of cognitive behavioural interventions that target the individual for reducing perfectionism (see [52], for a meta-analysis), enabling the perfectionistic individual to continue to manage their perfectionism over the long-term has proven difficult [53]. In contrast, interventions that target the psychosocial environment in which perfectionism develops and is maintained have been suggested as an alternative avenue that researchers and practitioners may wish to consider in managing perfectionism [5,54]. For example, interventions may wish to educate significant others (e.g., dancers’ teachers, directors, parents) about the creation of psychological environments that are empowering rather than disempowering [55] and do not include the various behaviours, practices, and relational styles that contribute to a perfectionistic climate [54].

## 7. Conclusions

Despite these limitations, the results of this research contribute to the literature by extending information about the link between perfectionism and indicators of well- and ill-being using a sample of dancers, suggesting that amotivation can be considered an important and significant variable in these relationships in the dance context. Our results provide additional empirical evidence that motivationally, socially-prescribed perfectionism is maladaptive, via an association with high amotivation of dancers, which in turn is related to low feelings of energy and high symptoms of burnout. Self-oriented perfectionism, instead, through its negative relationship with amotivation, is related to high feelings of energy and lower symptoms of burnout. The amotivation of young vocational dancers can, therefore, regulate both the seemingly positive and negative effects of the dancers’ perfectionism.

## Figures and Tables

**Table 1:** Descriptive statistics, reliabilities, and bivariate correlations for study variables.

Variables	Mean	*SD*	Alpha	1	2	3	4	5	6
1. SPP	5.09	1.08	0.78	-					
2. SOP	3.39	1.30	0.80	0.30 **	-				
3. Autonomous M	5.40	0.83	0.87	0.01	0.34 **	-			
4. Controlled M	3.81	1.10	0.73	0.35 **	0.16	0.50 **	-		
5. Amotivation	1.95	1.08	0.78	0.19 *	−0.21 *	−0.26 **	−0.03	-	
6. Subjective Vitality	5.37	1.12	0.88	−0.11	0.24 **	0.40 **	0.24 **	−0.43 **	-
7. Burnout	1.89	0.84	0.85	0.21 **	−0.19 *	−0.32 **	−0.06	0.71 **	−0.53 **

Note. SPP = socially prescribed perfectionism; SOP = self-oriented perfectionism; M = motivation. Range all variables = 1–7, except for burnout = 1–5. ** *p* < 0.01; * *p* < 0.05.

**Table 2:** Mediational effects of forms of motivation and amotivation in the relationship between dimensions of perfectionism and well-ill being.

		Auton M	Control M	AM	Subjective Vitality	Burnout	IE Subjective Vitality	IE Burnout
Models								
SPP		−0.06	0.28 **	0.23 **	−0.12	0.04	−0.05	0.08sig
Mediator Autonomous M					0.27 *	−0.09 ^†^	−0.02	0.01
Mediator Controlled M					0.16	−0.00	0.04	−0.00
Mediator Amotivation					−0.33 **	0.32 **	−0.08sig	0.07sig
*R*^2^ total model					0.30 **	0.53 **		
SOP		0.28 **	0.06	−0.29 **	0.13	−0.02	0.18sig	−0.12sig
Mediator Autonomous M					0.27 *	−0.09 ^†^	0.08	−0.03
Mediator Controlled M					0.16	−0.00	0.01	−0.00
Mediator Amotivation					−0.33 **	0.32 **	0.10sig	−0.09sig
*R*^2^ total model					0.30 **	0.53 **		

Note. SPP = socially prescribed perfectionism; SOP = self-oriented perfectionism; Auton = autonomous; Control = controlled; AM = amotivation; M = motivation; IE = indirect effect. ** *p* < 0.01, * *p* < 0.05, ^†^ *p* < 0.10, sig = indirect effect significant (confidence intervals do not include zero).

## References

[1] Laws H. (2005). Fit to Dance 2.

[2] Hill A.P., Mallinson-Howard S.H., Madigan D.J., Jowett G.E., Tenenbaum G., Eklund R.C. (2020). Perfectionism in sport, dance, and exercise: An extended review and reanalysis. Handbook of Sport Psychology.

[3] Hamilton L.H. (2003). Depression in dancers: Nobody’s perfect-but try to tell that to an overachiever. Dance Mag..

[4] Eusanio J., Thomson P., Jaque S. (2014). Perfectionism, shame, and self-concept in dancers: A mediation analysis. J. Dance Med. Sci..

[5] Hall H.K., Hill A.P., Appleton P.R., Roberts G.C., Treasure D. (2012). Perfectionism: A foundation for sporting excellence or an uneasy pathway toward purgatory?. Advances in Motivation in Sport and Exercise.

[6] Gaudreau P., Thompson A. (2010). Testing a 2× 2 model of dispositional perfectionism. Pers. Individ. Differ..

[7] Hewitt P.L., Flett G.L. (1991). Perfectionism in the self and social contexts: Conceptualization, assessment, and association with psychopathology. J. Pers. Soc. Psychol..

[8] Stoeber J., Otto K. (2006). Positive conceptions of perfectionism: Approaches, evidence and challenges. Pers. Soc. Psychol. Rev..

[9] Hill A.P., Appleton P.R. (2011). The predictive ability of the frequency of perfectionistic cognitions, self-oriented perfectionism, and socially prescribed perfectionism in relation to symptoms of burnout in youth rugby players. J. Sport Sci..

[10] Hill A.P., Stoeber J., Brown A., Appleton P.R. (2014). Team perfectionism and team performance: A prospective study. J. Sport Exerc. Psychol..

[11] Gaudreau P., Verner-Filion J. (2012). Dispositional perfectionism and well-being: A test of the 2× 2 model of perfectionism in the sport domain. Sport Exerc. Perform. Psychol..

[12] Hill A.P., Hall H.K., Appleton P.R., Kozub S.A. (2008). Perfectionism and burnout in junior elite soccer players: The mediating influence of unconditional self-acceptance. Psychol. Sport Exerc..

[13] Hill A.P., Hall H.K., Appleton P.R., Murray J.J. (2010). Perfectionism and burnout in canoe polo and kayak slalom athletes: The mediating influence of validation and growth-seeking. Sport Psychol..

[14] Hill A.P., Hall H.K., Appleton P.R. (2010). A comparative examination of the correlates of self-oriented perfectionism and conscientious achievement striving in male cricket academy players. Psychol. Sport Exerc..

[15] Curran T., Hill A.P., Jowett G.E., Mallinson-Howard S.H. (2014). The relationship between multidimensional perfectionism and passion in junior athletes. Int. J. Sport Psychol..

[16] Appleton P.R., Hall H.K., Hill A.P. (2009). Relations between multidimensional perfectionism and burnout in junior-elite male athletes. Psychol. Sport Exerc..

[17] Appleton P., Hill A.P. (2012). Perfectionism and athlete burnout in junior elite athletes: The mediating role of motivation regulations. J. Clin. Sport Psychol..

[18] Hill A.P., Madigan D.J. (2017). A short review of perfectionism in sport, dance and exercise: Out with the old, in with the 2 × 2. Curr. Opin. Psychol..

[19] Cumming J., Duda J.L. (2012). Profiles of perfectionism, body-related concerns, and indicators of psychological health in vocational dance students: An investigation of the 2 × 2 model of perfectionism. Psychol. Sport Exerc..

[20] Quested E., Cumming J., Duda J.L. (2014). Profiles of perfectionism, motivation, and self-evaluations among dancers: An extended analysis of Cumming and Duda (2012). Int. J. Sport Psychol..

[21] Nordin-Bates S.M., Raedeke T.D., Madigan D.J. (2017). Perfectionism, burnout, and motivation in dance: A replication and test of the 2 × 2 model of perfectionism. J. Dance Med. Sci..

[22] Deci E.L., Ryan R.M. (1985). Intrinsic Motivation and Self-Determination in Human Behavior.

[23] Deci E.L., Ryan R.M. (2000). The “what” and “why” of goal pursuits: Human needs and the self-determination of behavior. Psychol. Inq..

[24] Ryan R.M., Deci E.L. (2000). Self-determination theory and the facilitation of intrinsic motivation, social development, and wellbeing. Am. Psychol..

[25] Ryan R.M., Deci E.L., Hagger M.S., Chatzisarantis N.L.D. (2007). Active human nature: Self-determination theory and the promotion and maintenance of sport, exercise, and health. Intrinsic Motivation and Self-Determination in Exercise and Sport.

[26] Ryan R.M., Deci E.L. (2017). Self-Determination Theory: Basic Psychological Needs in Motivation, Development and Wellness.

[27] Balaguer I., Castillo I., Duda J.L., Quested E., Morales V. (2011). Predictores socio-contextuales y motivacionales de la intención de continuar participando: Un análisis desde la SDT en danza [Social-contextual and motivational predictors of intentions to continue participation: A test of SDT in dance]. Rev. Int. Cienc. Deporte.

[28] Quested E., Duda J.L. (2011). Perceived autonomy support, motivation regulations and the self-evaluative tendencies of student dancers. J. Dance Med. Sci..

[29] Stoeber J., Damian L.E., Madigan D.J., Stoeber J. (2018). Perfectionism: A motivational perspective. The Psychology of Perfectionism: Theory, Research, Applications.

[30] Vicent M., Sanmartín R., Vásconez-Rubio O., García-Fernández J.M. (2020). Perfectionism profiles and motivation to exercise based on self-determination theory. Int. J. Environ. Res. Public Health.

[31] Miquelon P., Vallerand R.J., Grouzet F.M., Cardinal G. (2005). Perfectionism, academic motivation, and psychological adjustment: An integrative model. Pers. Soc. Psychol. Bull..

[32] Gaudreau P., Antl S. (2008). Athletes broad dimensions of dispositional perfectionism: Examining changes in life satisfaction and the mediating role of sport-related motivation and coping. J. Sport Exerc. Psychol..

[33] McArdle S., Duda J.L. (2004). Exploring social-contextual correlates of perfectionism in adolescents: A multivariate perspective. Cognit. Ther. Res..

[34] Mouratidis A., Michou A. (2011). Perfectionism, self-determined motivation, and coping among adolescents. Psychol. Sport Exerc..

[35] Van Yperen N.W. (2006). A novel approach to assessing achievement goals in the context of the 2 x 2 framework: Identifying distinct profiles of individuals with different dominant achievement goals. Pers. Soc. Psychol. Bull..

[36] Jowett G.E., Hill A.P., Hall H.K., Curran T. (2013). Perfectionism and junior athlete burnout: The mediating role of autonomous and controlled motivation. Sport Exerc. Perform. Psychol..

[37] Madigan D.J., Stoeber J., Passfield L. (2016). Perfectionism and changes in athlete burnout over three months: Interactive effects of personal standards and evaluative concerns perfectionism. Psychol. Sport Exerc..

[38] De Bruin K.A.P., Bakker F.C., Oudejans R.R.D. (2009). Achievement goal theory and disordered eating: Relationship of disordered eating with goal orientations and motivational climate in female gymnasts and dancers. Psychol. Sport Exerc..

[39] Quested E., Duda J.L. (2009). Setting the stage: Social-environmental and motivational predictors of optimal training engagement. Perform. Res..

[40] Atienza F., Appleton P., Hall H.K., Castillo I., Balaguer I. (2020). Validation of the Spanish version of multidimensional inventory of perfectionism in young footballers. Cuad. Psicol. Deporte.

[41] Cox B.J., Enns M.W., Clara I.P. (2002). The multidimensional structure of perfectionism in clinically distressed and college student samples. Psychol. Assess..

[42] Balaguer I., Castillo I., Duda J.L. (2007). Propiedades psicométricas de la Escala de Motivación Deportiva en deportistas españoles [Psychometric properties of the Sports Motivation Scale in Spanish athletes]. Rev. Mex. Psicol..

[43] Pelletier L., Tuson K., Fortier M., Vallerand R., Briere N., Blais M. (1995). Toward a new measure of intrinsic motivation, extrinsic motivation, and amotivation in sports - the Sport Motivation Scale (SMS). J. Sport Exerc. Psychol..

[44] Castillo I., Tomás I., Balaguer I. (2017). The Spanish-version of the Subjective Vitality Scale: Psychometric properties and evidence of validity. Span. J. Psychol..

[45] Ryan R.M., Frederick C. (1997). On energy, personality, and health: Subjective vitality as a dynamic reflection of well-being. J. Pers..

[46] González L., Castillo I., García-Merita M.L., Balaguer I. (2015). Apoyo a la autonomía, satisfacción de las necesidades psicológicas y bienestar: Análisis de la invarianza de un modelo en futbolistas y bailarines [Autonomy support, psychological needs satisfaction and well-being: Invariance of a structural model in soccer players and dancers]. Rev. Psicol. Deporte.

[47] Balaguer I., González L., Fabra P., Castillo I., Mercé J., Duda J.L. (2012). Coaches’ interpersonal style, basic psychological needs and the well- and ill-being of young soccer players: A longitudinal analysis. J. Sport Sci..

[48] Raedeke T.D., Smith A.L. (2001). Development and preliminary validation of an athlete burnout measure. J. Sport Exerc. Psychol..

[49] Quested E., Duda J.L. (2011). Antecedents of burnout among elite dancers: A longitudinal test of basic needs theory. Psychol. Sport Exerc..

[50] Hayes A.F. (2013). Introduction to Mediation, Moderation, and Conditional Process Analysis: A Regression-Based Approach.

[51] Cohen J. (1992). A power primer. Psychol. Bull..

[52] Lloyd S., Schmidt U., Khondoker M., Tchanturia K. (2015). Can psychological interventions reduce perfectionism? A systematic review and meta-analysis. Behav. Cogn. Psychother..

[53] Hewitt P.L., Flett G.L., Mikail S.F. (2017). Perfectionism: A Relational Approach to Conceptualization, Assessment, and Treatment.

[54] Hill A.P., Grugan M.C. (2019). Introducing Perfectionistic Climate. Perspect. Early Child. Psychol. Educ..

[55] Duda J.L. (2013). The conceptual and empirical foundations of Empowering Coaching™: Setting the stage for the PAPA project. Int. J. Sport Exerc. Psychol..

